# Clinical Profile and Outcomes of Rheumatic Heart Disease Patients Undergoing Surgical Valve Procedures in Uganda

**DOI:** 10.5334/gh.1260

**Published:** 2023-11-14

**Authors:** Joselyn Rwebembera, Andrew Y. Chang, Samalie M. Kitooleko, Gloria Kaudha, Sarah de Loizaga, Miriam Nalule, Kenneth Ahabwe, Wanzhu Zhang, Emmy Okello, Pranava Sinha, Tom Mwambu, Craig Sable, Andrea Beaton, Chris T. Longenecker

**Affiliations:** 1 Uganda Heart Institute, Kampala, Uganda; 2 Department of Epidemiology and Population Health, Stanford University School of Medicine, USA; 3 Center for Innovation in Global Health, Stanford University, USA; 4 Stanford Cardiovascular Institute, Stanford University, USA; 5 Cincinnati Children’s Hospital Medical Center, The Heart Institute, Cincinnati, OH 45229, USA; 6 School of Medicine, University of Cincinnati, Cincinnati, OH 45229, USA; 7 Department of Internal Medicine, College of Health Sciences, Makerere University, Kampala, Uganda; 8 Pediatric Cardiology, Children’s National Hospital, Washington, District of Columbia, USA; 9 Department of Global Health and Division of Cardiology, University of Washington, Seattle, WA, USA

**Keywords:** Rheumatic Heart Disease (RHD), Valve, Surgery, Repair, Profile, Outcomes

## Abstract

**Background:**

Chronic valvular heart disease is a well-known, long-term complication of acute rheumatic fever (ARF), which remains a major public health problem in low- and middle-income countries. Access to surgical management remains limited. Outcomes of the minority proportion of patients that access surgery have not been described in Uganda.

**Objectives:**

To describe the volume and type of rheumatic heart disease (RHD) valvular interventions and the outcomes of operated patients in the Uganda RHD registry.

**Methods:**

We performed a retrospective cohort study of all valve surgery procedures identified in the Uganda RHD registry through December 2021.

**Results:**

Three hundred and sixty-seven surgical procedures were performed among 359 patients. More than half were among young (55.9% were ≤20 years of age), female (59.9%) patients. All patients were censored at 15 years. The median (IQR) follow up period was 43 (22,79) months. Nearly half of surgeries (46.9%) included interventions on multiple valves, and most valvular operations were replacements with mechanical prostheses (96.6%). Over 70% of the procedures were sponsored by charity organizations. The overall mortality of patients who underwent surgery was 13% (47/359), with over half of the mortalities occurring within the first year following surgery (27/47; 57.4%). Fifteen-year survival or freedom from re-operation was not significantly different between those receiving valve replacements and those receiving valve repair (log-rank p = 0.76).

**Conclusions:**

There has been increasing access to valve surgery among Ugandan patients with RHD. Post-operative survival is similar to regionally reported rates. The growing cohort of patients living with prosthetic valves necessitates national expansion and decentralization of post-operative care services. Major reliance on charity funding of surgery is unsustainable, thus calling for locally generated and controlled support mechanisms such as a national health insurance scheme. The central illustration (Figure 1) provides a summary of our findings and recommendations.

**Figure 1:**
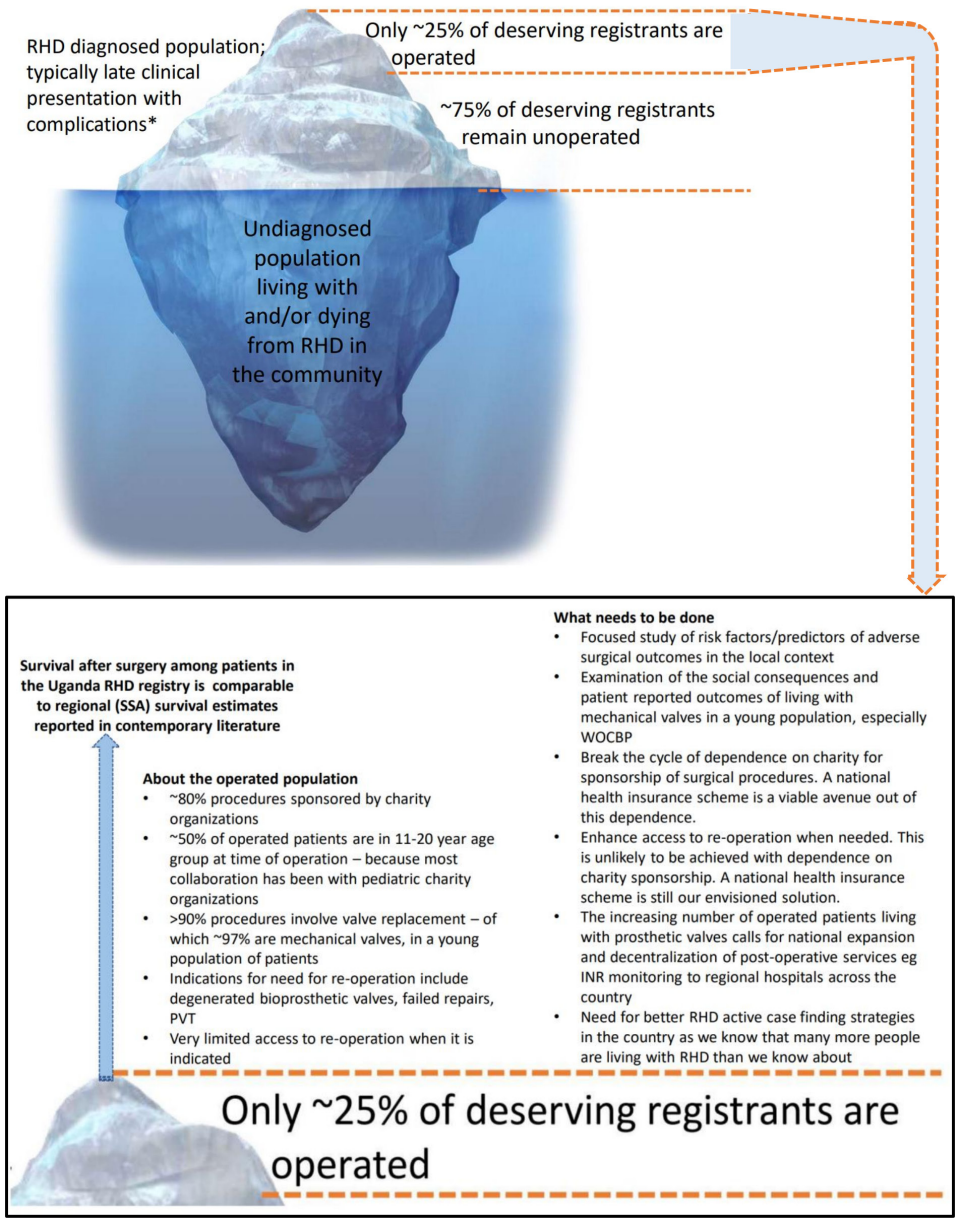
Central Illustration – General characteristics of surgical valve procedures in the Uganda national RHD registry and a needs assessment to increase access to surgical management of RHD in Uganda. *: Clinical complications of RHD include heart failure, arrhythmias, stroke and systemic embolism, infective endocarditis. RHD: Rheumatic Heart Disease; SSA: sub-Saharan Africa; PVT: Prosthetic Valve Thrombosis; WOCBP: Women of Child Bearing Potential; INR: International Normalized Ratio.

## Background

Regional [1] and national [2, 3] registries in Africa have revealed a high burden of un-operated rheumatic heart disease (RHD), which bears substantial consequences including recurrent hospitalizations and premature deaths. Although access to interventional cardiac procedures is slowly increasing, multi-sectoral barriers remain [4, 5]. In addition to increasing interventional volume and access, ensuring good outcomes is critical. Few studies have described good-to-excellent immediate and short-term outcomes following valve surgery for RHD in sub-Saharan Africa [6,7,8,9,10]. Additional studies encompassing longer-term outcomes are needed to inform local and regional care and investment in tertiary management of RHD.

Uganda provides a case study of establishing sustainable RHD tertiary care services in sub-Saharan Africa. Cardiac surgery with cardiopulmonary bypass commenced in 2007 at the Uganda Heart Institute (UHI), but capacity was limited. Foreign teams were coordinated by Children’s National Hospital, Washington DC; Gift of Life International, New York; Edwards Life Sciences/Thoracic Surgery Foundation and Samaritan’s Purse Children’s Heart Project, Washington DC. They offered financing, capacity building consultation, skill-transfer and on-ground support [11]. Today, the local Ugandan team has built capacity to perform complex congenital heart and valve surgeries with survival rates after open heart surgery (OHS) being comparable between independent and mentored procedures. [11, 12] The growth in clinical capacity and infrastructure has thus improved access to cardiovascular interventions in Uganda.

The Uganda National RHD registry was conceived in 2009 with an over-arching aim of streamlining RHD care in the country. Patients who were already in clinical care with a diagnosis of RHD, mostly un-operated, and a few who had undergone valve surgery from various centers globally were enrolled at the registry’s inception. Since then, all patients with new RHD clinical presentations are enrolled immediately. This longitudinal database captures clinical information from all known people living with RHD in the country who seek care from UHI as well as the four regional centers of excellence in Lubowa (central), Mbarara (west), Gulu (north), and Lira (northeast) [13]. In this paper, we aimed to summarize the volume and outcomes of cardiac valve surgical procedures among participants in the Uganda National RHD registry.

## Methods

We conducted a retrospective descriptive cohort study of the Uganda RHD registry database for all participants with RHD who had a surgical valve intervention procedure. This included patients who had been operated upon prior to registry inception (2009) and those who were operated on while the registry was running, through December 31, 2021. All subjects who agreed to participate in the registry provided written informed consent (if age 18 years or older), parental consent (if under 18 years), and additional participant assent if aged eight-17 years [14]. The study was approved by the institutional review board of Makerere University School of Medicine (Mak-SOMREC-2013-072) and the Uganda National Council for Science and Technology.

A structured form was used to extract demographic and clinical data from the registry database. Among those who had a surgical intervention, major clinical events such as prosthetic valve thrombosis, endocarditis, re-operation, or death were captured. To supplement registry data, in-patient and out-patient medical records were reviewed for data that were missing from the registry. Telephone calls were used to update patient survival status and complications. Surgical procedures were defined as any valve repair, replacement or open commissurotomy. Patients with severe RHD on echocardiography (any combination of: severe mitral regurgitation, severe aortic regurgitation, and/or moderate/severe mitral stenosis) and any symptoms *or* patients with moderate RHD on echo (any combination of: moderate mitral regurgitation and/or moderate aortic regurgitation) and severe symptoms (NYHA Class IV) were considered to need surgery. Surgical mortality was defined as death prior to hospital discharge OR within 30 days of surgery; while late mortality was defined as death after hospital discharge OR after 30 days of surgery.

The initial records in the database were collected using paper forms and then transcribed using the REDCap (Nashville, TN, USA) data management platform [15, 16]. Statistical analyses were performed using the Stata 12 computational suite (College Station, TX, USA) as well as Microsoft Excel (Redmond, WA, USA). Descriptive findings were summarized with counts and proportions. Kaplan Meier survival curves and log-rank testing were used to assess survival and freedom from re-operation between types of intervention (repair vs. replacement).

## Results

### Types of surgical interventions

During the timeframe of the study, 3,059 patients were enrolled in the registry. Of these, 2,645 were enrolled for clinical RHD while 414 were enrolled for latent RHD (borderline or mild definite RHD found on echo screening). Slightly more than half (1,510; 57% patients) met criteria for needing surgery.

There were 367 surgical valve procedures identified among 359 patients. More than half (59.9%) were female and nearly 80% had undergone the operation at age 30 or younger (Table 1). All patients were censored at 15 years. The median (IQR) follow up time was 43 (22,79) months.

**Table 1:** General characteristics of surgical valve procedures.


VOLUME OF PROCEDURES	

Total number of procedures	367

Total number of patients	359

Female (n = 359 patients)	215 (59.9%)

**AGE AT TIME OF SURGERY, YEARS (n = 367 PROCEDURES)**	

5–10	25 (6.8%)

11–20	180 (49.1%)

21–30	79 (21.5%)

31–40	44 (12.0%)

41–50	31 (8.4%)

51–60	5 (1.4%)

61–70	3 (0.8%)

**TYPE OF SURGICAL PROCEDURE (n = 367)**	

MV Replacement	127 (34.6%)

MV Replacement and TV Repair	68 (18.5%)

MV and AV Replacement	57 (15.5%)

MV and AV Replacement, TV Repair	33 (9.0%)

AV Replacement	33 (9.0%)

MV Repair	31 (8.5%)

MV Repair and TV Repair	9 (2.5%)

AV Replacement and MV Repair	4 (1.1%)

AV Repair	2 (0.5%)

Mitral Commissurotomy	2 (0.5%)

MV, AV, and TV Replacement	1 (0.3%)

**TYPES OF PROSTHETIC VALVES USED IN VALVE REPLACEMENT PROCEDURES (n = 323 PROCEDURES)**	

Mechanical Valve	312 (96.6%)

Bioprosthetic Valve	11 (3.4%)

**SURVIVAL STATUS (n = 359 PATIENTS)**	

Alive	307 (85.5%)

Dead	47 (13.1%)

Unknown Status**‡**	5 (1.4%)

**SPONSORING ENTITY (n = 367 PROCEDURES)**	

Charity Efforts	286 (77.9%)

Patient/Family Out-of-Pocket Payment	69 (18.8%)

Employer	10 (2.7%)

Medical Insurance	2 (0.5%)

**COUNTRIES (AND CENTERS) WHERE SURGERY WAS PERFORMED (n = 367 PROCEDURES)**	

Uganda (Uganda Heart Institute)	115 (31.4%)

Sudan (Salaam Center)	188 (51.2%)

India (multiple centers)	46 (12.5%)

Other Countries	18 (4.9%)


MV: Mitral valve; TV: Tricuspid Valve; AV: Aortic valve; **‡**Unknown status was assigned to patients who had not been seen in any RHD registry clinic for more than 1 year and had been unreachable on phone for more than 6 months.

Nearly all procedures (90.4%) involved the mitral valve, with the most common surgery being isolated mitral valve replacement (127/367; 34.6%). Only 35 (9.5%) isolated aortic valve procedures were registered. Nearly one-third of operations (138/367; 37.6%) addressed two valves, and about one in ten (44/367; 12.0%) addressed three valves. The vast majority of surgeries (323/367; 88%) involved at least one valve replacement, and nearly all of these (312/323; 96.6%) were mechanical valves.

The majority of operations (286/367; 77.9%) were sponsored by charity efforts and the fewest covered by medical insurance (2/367; 0.5%).

Nearly all procedures performed outside of Uganda were sponsored by charity efforts. For procedures in Uganda, approximately half were sponsored by charity efforts while the other half were financed by patient/family out-of-pocket payments and other funding mechanisms.

### Surgical volumes and distribution

**Figure 2:**
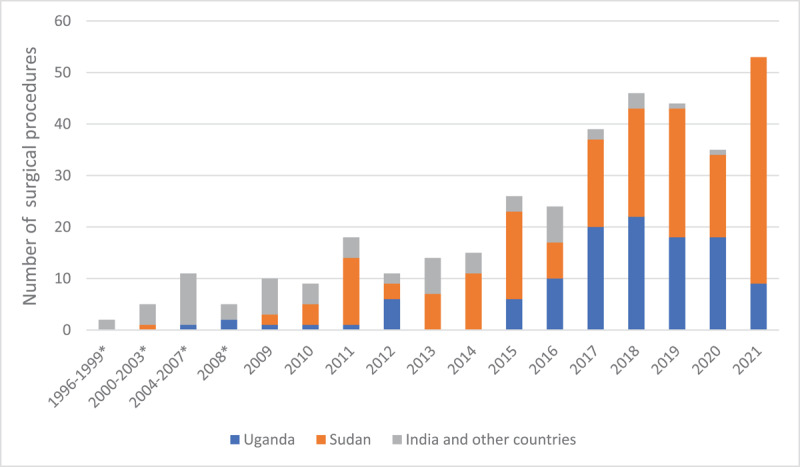
Number of surgical procedures by year and country where they were performed. *Patients were operated upon before inception of the Uganda national RHD registry, but were enrolled into the registry upon its inception in 2009. They continue to receive care under the registry with all clinical outcomes registered prospectively.

The volume of valve surgeries generally increased over time, with a growing proportion undertaken at UHI and the Salam Center in Sudan. The exception was in 2020, due to the impact of the SARS-CoV-2 (COVID-19) pandemic (Figure 2). UHI volumes dropped starting in 2018 during a scheduled renovation of the UHI wards and surgical intensive care unit that was still ongoing by the end of the study period.

### Mortality after surgical procedures

The overall mortality of patients who had undergone surgery was 13% (47/359), with 45 deaths occurring post valve replacement and two occurring post valve repair procedures. Of the 47 confirmed mortalities, 27 (57%) occurred within one year of operation, and nearly two-thirds occurred within the first two years post-operatively (Table 2). Fatalities that occurred during the surgical hospitalization were most often attributed to cardiac and/or multi-organ failure while those that happened after discharge from the surgical hospitalization were largely attributed to cardiac failure. The cause of death could not be ascertained in a significant proportion of patients who died after discharge from hospital. Mortality was similar among patients who were operated on at UHI as compared to outside the country. Only five (1.4%) patients had unknown survival status.

**Table 2:** Description of known deaths following valve replacement surgery.


	MORTALITY AFTER VALVE REPLACEMENT (n = 45)

Females, n (%)	27 (60%)

Patients with mechanical valves, n (%)	44 (98%)

Patients with bioprosthetic valves, n (%)	1 (2%)

**Time from operation to death**, n (%)	

Surgical mortality*	14 (31%)

Late mortality**#**	

• Within 12 months	13 (29%)

• 13–24 months	5 (11%)

• 25–36 months	5 (11%)

• >36 months	8 (18%)

**Causes of death¶**	

Surgical mortality*, n (%)	14 (31%)

• *Post-operative hemorrhagic shock*	*1*

• *Post-operative cardiac and/or multi-organ-failure*	*13*

Late mortality**#**, n (%)	31 (69%)

• *Heart Failure*	*10*

• *Major Bleeding (VKA-related)*	*2*

• *Confirmed prosthetic valve thrombosis*	*2*

• *Suspected prosthetic valve thrombosis*	*2*

• *Venous thrombo-embolism*	*1*

• *Acute reaction to BPG*	*1*

• *Acute Kidney Injury*	*1*

• *Severe Malaria*	*1*

• *Homicide*	*1*

• *Sudden unexpected death**†***	*4*

• *Unknown (unclear or total lack of history surrounding the death)**‡***	*6*


BPG: Benzyl Benzathine Penicillin; *Surgical mortality: death prior to hospital discharge OR within 30 days of surgery; **#**Late mortality: death after hospital discharge OR after 30 days of surgery; **¶**Causes of death: most causes of death were confirmed from hospital records; ***†***Sudden unexpected death based on verbal autopsy with a family member; ***‡***Verbal autopsy attempted but unfruitful or inconclusive.

Nearly a quarter of procedures (91; 24.7%) were double valve replacements. Outcomes in this group of patients by country where procedures were performed are shown in Table 3.

**Table 3:** Mortality following double valve replacement and other surgical procedures by country where operations were performed.


	DOUBLE VALVE REPLACEMENT‡			OTHER SURGICAL PROCEDURES¶		
	
SURGICAL MORTALITY*	**UGANDA (n = 26)**	**ABROAD (n = 65)**	**P-VALUE**	**UGANDA (n = 89)**	**ABROAD (n = 187)**	**P-VALUE**

	2 (7.7%)	2 (3.1%)	0.686	7 (7.9%)	3 (1.6%)	0.024

**Late Mortality#**	**UGANDA (n = 24)**	**ABROAD (n = 63)**	**P-VALUE**	**UGANDA (n = 82)**	**ABROAD (n = 184)**	**P-VALUE**

	4 (16.7%)	7 (11.1%)	0.486	7 (8.5%)	15 (8.2%)	0.916


* Surgical mortality: death prior to hospital discharge OR within 30 days of surgery; #Late mortality: death after hospital discharge OR after 30 days of surgery; ‡‘Double valve replacement procedures’ included replacement of the mitral and aortic valve, with or without tricuspid valve intervention; ¶‘Other surgical procedures’ included single valve replacement procedures with or without repair, valve repair-only procedures, and surgical commissurotomy procedures.

### Survival and freedom from re-operation

**Figure 3:**
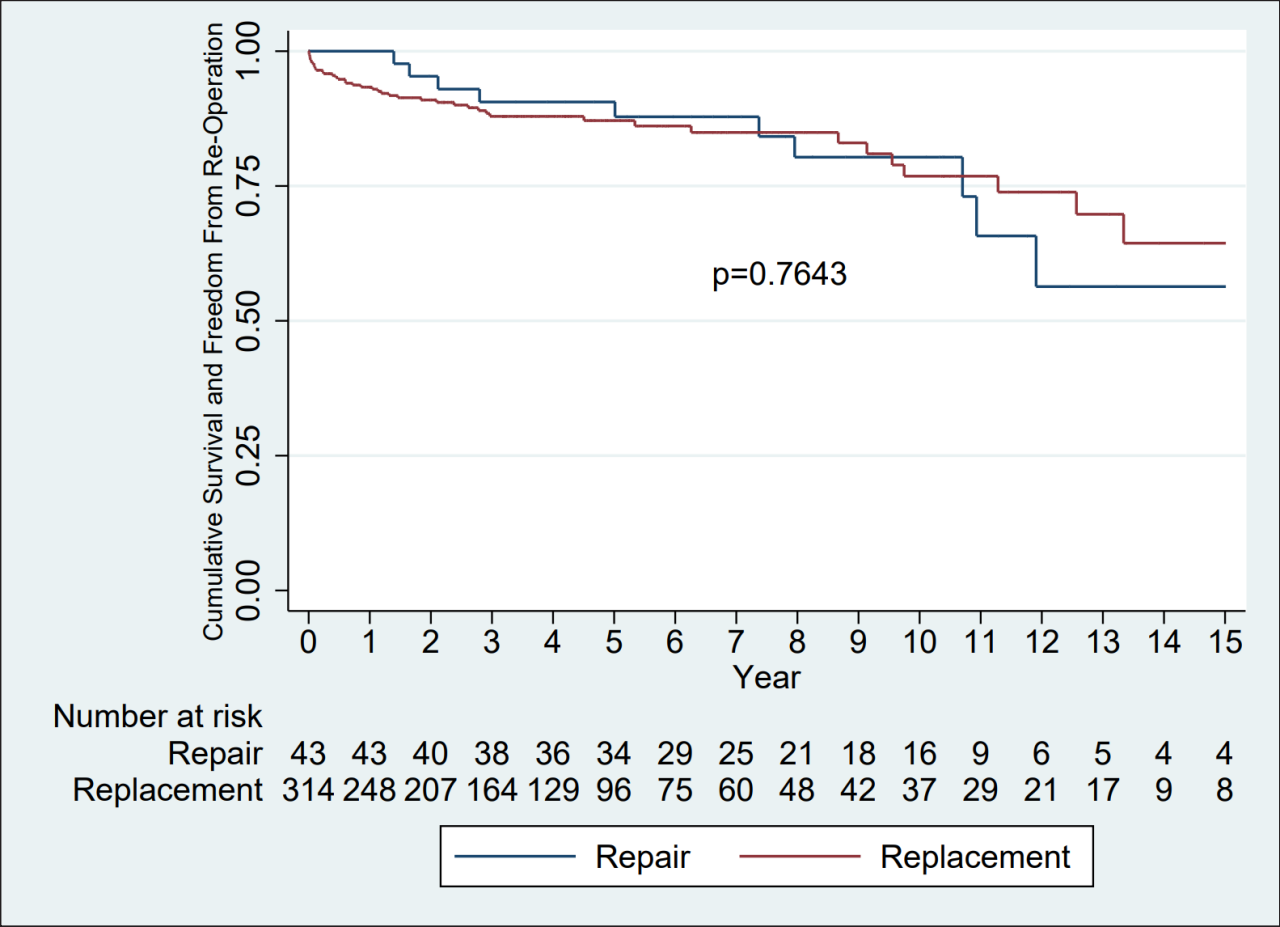
Kaplan-Meier estimates of survival or freedom from re-operation in registry patients undergoing valve replacement versus valve repair. Valve repair and mitral commissurotomy, n = 43 (41 valve repairs, 2 mitral valve commissurotomies); Valve replacement, n = 314. This analysis excluded 1 valve repair and 4 valve replacement patients who had unknown outcomes.

We next compared the outcomes (re-operation or death) of registry patients who underwent valve repair or commissurotomy with those who received valve replacement operations. ‘Valve repair’ included mitral and/or aortic valve repair procedures with or without tricuspid valve repair, and without concurrent valve replacement. ‘Commissurotomy’ included open mitral valve commissurotomy procedures. ‘Valve replacement’ included mitral and/or aortic valve replacement (with metallic or bioprosthetic valves) with or without repair of another valve. Only index procedures were included in this analysis. Kaplan-Meier statistics did not reveal a statistically significant difference in a composite outcome of death or freedom from re-operation at 15 years of follow-up (p = 0.7643) (Figure 3).

### Complications/other outcomes

#### Mechanical valves

Among the 312 patients who received mechanical valves, we identified four cases of prosthetic valve thrombosis (PVT) (two cases confirmed on imaging, two cases suspected on clinical grounds), resulting in 100% fatality (Table 2). The two confirmed cases occurred at 171- and 1,604-days post-surgery. Both were managed with thrombolytic therapy which was unsuccessful, and surgical intervention was not readily available. The two suspected cases occurred at 42- and 403-days post-surgery, and both died before reaching the tertiary center for confirmatory investigations to be conducted. There was no record of mechanical valve endocarditis.

#### Bioprosthetic valves

Among the 11 patients who received bioprosthetic valves, six (54.5%) experienced valvular degeneration requiring re-operation. Two later received a mechanical valve, two died from heart failure while waiting, and two were alive on chronic management of heart failure at the time of this audit. There was no record of bioprosthetic valve endocarditis.

#### Mitral and/or aortic valve repair

Forty-five patients had mitral and/or aortic valve repair procedures. Eighteen (39.1%) of the 45 patients had documented valvular dysfunction necessitating re-operation, although only five accessed the required surgical re-intervention. By the time of the audit, two had died while waiting, and 11 were alive but on therapy for chronic heart failure secondary to failed valve repairs.

#### Overall need for re-operation in the registry

In total, 32 patients had documented indication for re-operation after their first surgical procedures (recurrence of mitral and/or aortic valve dysfunction after repair – 18, degeneration of bioprosthetic valves – 6, prosthetic valve thrombosis – 4, recurrence of mitral valve dysfunction after commissurotomy – 2, annular calcification causing severe aortic stenosis after AV replacement – 1, and aortic valve patient-prosthetic-mismatch – 1). Only eight (25%) of the 32 patients had been re-operated at the time of this audit, seven (22%) had died before accessing the repeat procedures, and 17 (53%) were on charity surgery waiting lists.

## Discussion

Our study represents the first dedicated analysis of valvular surgery outcomes among people living with RHD in Uganda and adds to knowledge already contributed by the few previous studies that presented follow-up clinical outcomes of such patients in sub-Saharan Africa [6,7,8]. The vast majority of operations in our cohort involved the mitral valve, but nearly half of surgeries included interventions on multiple valves. Most valvular operations were mechanical valve replacements despite the very juvenile patient population. This decision is likely driven by the ‘once in a lifetime opportunity’ to access valve surgery that is sponsored by charity. The longer-term impact of this commitment, especially among women of child bearing age in the local context, needs to be further examined.

In our study, the 11 to 20-year old age group formed nearly half of the total population of operated patients. In the natural history of RHD, clinical disease peaks in the 20–30 year age group [17]. Predominance of the 11–20 years among the operated population in our registry is likely due to the fact that financing of most of UHI’s surgical patient population comes from collaborations with pediatric charity organizations. Other patterns such as predominance of females and the most commonly affected valves in our cohort are in keeping with the known epidemiology of RHD in the region [17, 18].

Until 2009, provision of surgical cardiac procedures in Ugandans was predominated by centers in India. However, from 2010, we have witnessed a concentrated provision of procedures in Uganda and Sudan. The Salam Center for Cardiac Surgery in Sudan is a humanitarian project run by the NGO EMERGENCY [19]. This center has continued to offer immense surgical support to the RHD population in Uganda, with the number of surgical procedures superseding those performed in Uganda. The procedures performed at this institution are completely free to patients and the mission additionally pays for patients’ return air fare. In contrast, over half of the procedures performed in Uganda are covered by personal or family out-of-pocket payments. The Salam center and other aforementioned charity organizations have greatly increased access to surgery for the Ugandan RHD patient population. These missions collaborate with the Uganda National RHD registry for patient selection and preparation for surgery, and for post-operative intermediate-to-long-term care. Nevertheless, in general, the outcomes of patients operated upon at UHI were similar to those operated on outside the country.

Mortality data confirmed death in 13.1% of operated patients, which is within the range that is reported in the contemporary RHD valvular outcomes literature [20, 8, 21, 22]. This rate is also similar to regionally reported outcomes [6,7,8]. The predominance of heart failure as the reported cause of death in the first, second, and third post-operative years has similarly been reported in neighboring Rwanda [8]. The study in neighboring Tanzania, which explored factors associated with 30-day mortality, found double valve replacement as the only independent predictor of early mortality. While our analysis did not set out to assess factors associated with mortality, we hypothesize that severely delayed access to surgery with irreversible myocardial remodeling continues to drive heart failure even after valve intervention. We also note that the mortality for all patients receiving surgery is much higher than that reported for the pediatric-only cohort from the same dataset [3]. This implies that adult patients have worse outcomes post-operatively, which may in part be explained by surgical charity missions focusing on the pediatric population - and adult patients who mostly pay out-of-pocket having more advanced disease by the time they access surgery. These hypotheses require further exploration with a dedicated risk factor study.

Access to medically indicated re-operations was limited – not an unexpected phenomenon in settings with lengthy waiting lists where the bulk of procedures are sponsored by charity efforts. The consequences of this phenomenon have been observed in a similar setting in Rwanda, where 50% of the mortalities were due to bioprosthetic valve degeneration [8]. Although there were few events in our study, PVT carried a 100% rate of fatality despite thrombolytic therapy, indicating the critical need to maintain therapeutic levels of anticoagulation in our setting where opportunities to access emergency or even elective re-operation are dismal.

We also found that valve replacement and valve repair patients experience similar outcomes with regard to survival and freedom from reoperation. As opposed to the superior outcomes of mitral valve repair in the spectrum of mitral valve diseases, valve repair in rheumatic mitral valve disease has continued to have mixed outcomes especially due to the compromise in durability [21,22,23]. Our data lend support to the current guidelines which recommend consideration of repair of rheumatic valves only if a durable and successful repair is likely or when the individual advisability of long-term anticoagulation management is undesirable [24]. Additionally, the likelihood of successful repair is dependent on surgical experience and frequency of cases [25], which has been a considerable challenge at UHI.

The largest bulk of procedures were sponsored by NGOs and international collaborations; very few were supported by family out-of-pocket payments, health insurance or employers. Regrettably, such financing mechanisms frequently lead to long wait times and delays in care, both with negative health impacts – exemplified by the subset of patients with documented indication for re-operation where 22% of patients died because of failure to access re-operation and 53% still-standing on a lengthy charity waiting list. This evidence states the urgent need for all-inclusive and sustainable healthcare system financing plans, such as a national healthcare insurance scheme. Locally generated funding strategies to ensure universal health coverage for patients living with RHD are not a matter of debate, but of national importance [26].

We know a large number of deserving RHD patients in the Uganda national RHD registry remain unoperated [2, 3, 18]. Although not the primary aim of this study, we estimated that only a quarter of patients in the RHD registry who needed a surgical intervention received it. The overall number of patients in need of RHD surgery in Uganda is likely much higher, given that a minority of the Ugandan population with RHD is represented in the National RHD registry. While this study was not designed to assess mortality and morbidity among those who did not receive surgery, it is likely that the outcomes in adults living with RHD who are waiting for surgery would be no better than what we have recently reported for unoperated children in the Uganda RHD registry [3]. The five-year mortality for children with clinical RHD who did not receive intervention was > 40%, with a median time from diagnosis to death of eight months [3]. Similarly, the REMEDY study reported a two-year mortality of 20% [18] and that for those living with RHD in low income countries, only 11% and 1% of patients had access to surgery and catheterization, respectively [1].

To ensure timely operative management, the expansion of surgical facilities at UHI and decentralization of cardiac medical and surgical services for RHD care to regional referral hospitals across the country is not a matter of debate, but of national importance. This implies investment in systems, structures, equipment and, critically, human resource development. A dynamic mobile application to enable community-based decentralization of RHD case management in Uganda is being piloted in Lira and Gulu districts (protocol manuscript under review). If it proves effective, the government could adopt it to transform RHD care across the country.

There is a need for better RHD screening strategies in the country because many more people are living with RHD than we know about. In this regard, our group is piloting the *“Accelerating Delivery of rheUmatic heart disease preventive iNterventions in Uganda” (*ADUNU) program, a package of interventions built around evidence-based practices for RHD designed to be delivered within the public healthcare system in Uganda, including community-based active case-finding by echocardiography.

Our findings and recommendations for surgical management of RHD as part of the tertiary prevention strategy are summarized in the central illustration (Figure 1).

## Limitations

Our study was limited by its retrospective nature with resultant missing clinical follow-up information. The lack of timely echocardiographic diagnostics at the district and some regional hospitals may have led to misclassification of the outcome regarding cause of death as simply ‘heart failure’. Additionally, significant gaps in demographic and clinical (e.g., pre-operative echocardiographic parameters) information precluded risk factor analysis.

## Conclusions and future research directions

There has been increasing access to valve surgery among patients in the Ugandan National RHD Registry over the past two decades. Survival among patients in this registry after valve surgery is comparable to regionally reported rates. The growing cohort of patients living with prosthetic valves urges national expansion of post-operative care services. Critically, with the already existing facilities at UHI, cases must be performed frequently enough to leverage economies of scale for personnel and supplies.
